# Recommendations for core critical care ultrasound competencies as a part of specialist training in multidisciplinary intensive care: a framework proposed by the European Society of Intensive Care Medicine (ESICM)

**DOI:** 10.1186/s13054-020-03099-8

**Published:** 2020-07-03

**Authors:** Adrian Wong, Laura Galarza, Lui Forni, Daniel De Backer, Michael Slama, Bernard Cholley, Paul Mayo, Anthony McLean, Antoine Vieillard-Baron, Daniel Lichtenstein, Giovanni Volpicelli, Robert Arntfield, Ignacio Martin-Loeches, Gizella Melania Istrate, František Duška, on behalf of ESICM Critical Care Ultrasound Group, Adrian Wong, Laura Galarza, Lui Forni, Daniel De Backer, Michael Slama, Bernard Cholley, Paul Mayo, Anthony McLean, Antoine Vieillard-Baron, Daniel Lichtenstein, Giovanni Volpicelli, Robert Arntfield, Ignacio Martin-Loeches, Gizella Melania Istrate, František Duška

**Affiliations:** 1 European Society of Intensive Care Medicine, Brussels, Belgium; 2 Department of Critical Care, King’s College Hospital, London, UK; 3 Department of Intensive Care, Hospital General Universitario de Castellon, Castellon de la Plana, Spain; 4 Department of Intensive Care, Royal Surrey County Hospital, Guildford, UK; 5 Department of Intensive Care, CHIREC Hospitals, Université Libre de Bruxelles, Brussels, Belgium; 6 Medical Intensive Care, DRIME department in University Hospital of Amiens, Amiens, France; 7 Service d’Anesthésie-Réanimation, Hôspital Européen Georges Pompidou, AP-HP, Paris, France; 8 Division of Pulmonary, Critical Care, and Sleep Medicine, Northshore/Long Island Jewish Medical Centers, Northwell Health, New Hyde Park, NY USA; 9 Department of Intensive Care Medicine, Nepean Hospital, University of Sydney, Sydney, Australia; 10 Unit of Medical-Surgical Intensive Care, Ambroise Paré University Hospital, Assistance Publique-Hôpitaux de Paris, Boulogne-Billancourt, France; 11 Medical Intensive Care Unit, Hospital Ambroise-Paré (AP-HP), Boulogne (Paris-Ouest university), Boulogne-Billancourt, France; 12 Department of Emergency Medicine, San Luigi Gonzaga University Hospital, Orbassano, Torino, Italy; 13 Division of Critical Care, Department of Medicine, Western University London, Ontario, Canada; 14 St James’s Hospital, Multidisciplinary Intensive Care Research Organization (MICRO), Dublin, Ireland; 15 Department of Anaesthesia and Intensive Care, Charles University, Third Faculty of Medicine and FNKV University Hospital in Prague, Prague, Czech Republic

**Keywords:** Core critical care ultrasound, Specialist training, Competencies, Education in intensive care

## Abstract

**Abstract:**

Critical care ultrasound (CCUS) is an essential component of intensive care practice. Although existing international guidelines have focused on training principles and determining competency in CCUS, few countries have managed to operationalize this guidance into an accessible, well-structured programme for clinicians training in multidisciplinary intensive care. We seek to update and reaffirm appropriate CCUS scope so that it may be integrated into the international Competency-based Training in Intensive Care Medicine. The resulting recommendations offer the most contemporary and evolved set of core CCUS competencies for an intensive care clinician yet described. Importantly, we discuss the rationale for inclusion but also exclusion of competencies listed.

**Background/aim:**

Critical care ultrasound (CCUS) is an essential component of intensive care practice. The purpose of this consensus document is to determine those CCUS competencies that should be a mandatory part of training in multidisciplinary intensive care.

**Methods:**

A three-round Delphi method followed by face-to-face meeting among 32 CCUS experts nominated by the European Society of Intensive Care Medicine. Agreement of at least 90% of experts was needed in order to enlist a competency as mandatory.

**Results:**

The final list of competencies includes 15 echocardiographic, 5 thoracic, 4 abdominal, deep vein thrombosis diagnosis and central venous access aid.

**Conclusion:**

The resulting recommendations offer the most contemporary and evolved set of core CCUS competencies for an intensive care clinician yet described.

## Introduction

In 2009, the CHEST journal published the ACCP/SRLF Statement on Competency in Critical Care Ultrasonography [1]. This was a cooperative project between French and North American colleagues that led to an additional document that was published in 2011 titled Training Standards for Critical Care Ultrasonography. The training statement was prepared and approved by a working group of 22 professional societies from around the world including major societies from North America [2]. The competency statement was adopted as the foundation document for the training statement. A similar working group was brought together in 2014 and formulated the training statement on Competency in Advanced Critical Care Echocardiography (ACCE). This statement used the principles established in the ACCP/SRLF statement and forms the basis for the certification in ACCE that is now available in North America and Europe [3].

Critical care ultrasound (CCUS) is an essential component of intensive care practice. Although existing international guidelines have focused on training principles and determining competency in CCUS [1–3], few countries have managed to operationalize this guidance into an accessible, well-structured programme for clinicians training in multidisciplinary intensive care [4, 5]. It is thus incumbent upon CCUS leaders to review existing competencies specifically with the purpose of informing robust national training programmes within the framework defined by the European Union of Medical Specialists [5] and clarify any confusion that may have arisen since the initial guidelines were launched almost a decade ago.

We aimed to address this need by clearly defining the required competencies, so that they may be integrated into critical care function. The goal of this document is to provide specific guidance to the international critical care community by establishing unambiguous standards for training and competency in CCUS. The primary criterion is that all core competencies need to be of clinical value in the general intensive care setting. A further consideration is that these competencies need to be deliverable by trainers across a wide range of critical care settings.

## Methods

Following a systematic review of international CCUS training schemes [3], ESICM representatives (AW, LG, FD) approached the corresponding authors of the existing guidelines [1, 3] to form a core group of 15 experts, including 2 educators, a trainee and a consultant educationalist. Subsequently, members of relevant ESICM subcommittees and CCUS experts nominated by ESICM Council national representatives were invited to form an extended group of 17 additional experts. The combined (core and extended) CCUS group of experts and action plan were endorsed by both the ESICM Executive Committee and CoBaTrICe Committee (Competency-Based Training programme in Intensive Care Medicine for Europe).

A modified Delphi exercise was performed using web-based questionnaires and a final face-to-face round between June and September 2019 [6]. The Delphi technique was chosen as it allowed the exploration of a field beyond existing knowledge, discussion among experts and formation of consensus; the modified version was chosen because it allowed for expert interaction in the final round. The questionnaire was designed with questions based on a 5-point Likert scale [7], ranging from ‘strongly disagree’ to ‘strongly agree’, without a default answer setting to avoid influencing the experts’ responses. Questions were organized by domains per page and with nearly any matrix to minimize straightlining phenomenon. Throughout the whole process, the answers were evaluated by AW, LG and FD to identify inconsistencies in response patterns of individual members or heterogeneous answers. In these cases, members were contacted to ascertain their understanding of the question and confirm that it had not been an error during questionnaire completion.

The first exploratory survey was conducted within the core group to evaluate the intelligibility of the questions and completeness of the questionnaire. The first round with the combined group included closed questions and a free-text response within each domain, providing the opportunity to elaborate their responses. The second round included the original statements plus those derived from the free-text answers in the first round. This was supplemented by a face-to-face meeting of the core group.

After each round, we calculated basic descriptive statistics (median and IQR) for each statement; a summary of the survey results was sent to the combined group. In order to provide a recommendation, the combined group a priori agreed that the degree of required consensus be > 80% of agreement threshold. All competencies were further reviewed during the face-to-face meeting with the goal of reaching an agreement level of > 90%.

**Fig. 1:**
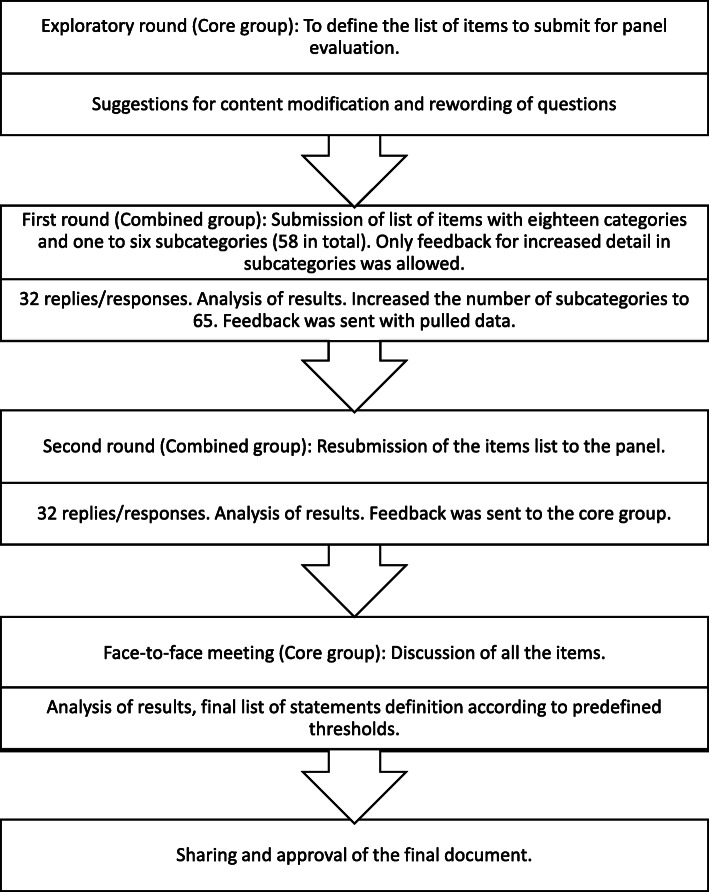
Flowchart summarizing the steps of the process

The steps of the process are summarized in Fig. 1.

## Results

Thirty-two selected experts were invited and agreed to participate in this Delphi exercise, all of whom completed the exercise. We had a response rate of 100% for all questions in all rounds. Thirteen experts attended the face-to-face meeting, with the remaining two unable to attend due to conflicting obligations. A full list of experts is provided in Supplementary material 1.

In the exploratory round, six domains and fifty-eight statements were proposed. Minor changes regarding completeness were made. In the first round, we were able to obtain consensus in 27 statements. All free-text comments were incorporated into the questionnaire; more than 75% of comments were duplicated. Based on free-text answers, we increased the statements to 65 in total. In the second round, we increased the number of statements with consensus to 32. During the face-to-face meeting, we were able to attain consensus on 53 statements and we were able to agree on a final list of competencies. Tables 1 and 2 contain the final results of the exercise with the whole statements explored.

**Table 1:** Summary of Delphi exercise

Domains	Number of statements in each domain			Proportion of statements where consensus was achieved		
	Round 1	Round 2	Face-to-face	Round 1 (%)	Round 2 (%)	Face-to-face (%)
Echocardiography	31	36	36	51.6	55.6	83.3
Thoracic	6	6	6	83.3	83.3	83.3
Diaphragm	3	4	4	0	0	50
Abdominal	6	7	7	33.3	33.3	85.7
Vascular	6	6	6	66.6	83.3	83.3
Other modalities	6	6	6	0	0	83.3
Totals	58	65	65	46.6	49.2	81.5

Consensus was achieved when 80% of the participants strongly agreed/agreed or strongly disagreed/disagreed with a statement in round 1 and 2. In face-to-face meeting, consensus was achieved when 90% of participants agreed or disagreed

**Table 2:** Results of the Delphi process with the full competencies explored

	Agreement		No agreement
	Include	Not to include	
**Echocardiography**			
Syndromes	Severe hypovolemia LV failure RV failure Tamponade Acute cor pulmonale Severe valvular abnormalities	Post-cardiac arrest management*	
Left ventricle	Size (qualitative) Systolic function (qualitative) Contraction pattern (qualitative) Valvular disease (qualitative: colour doppler)	Systolic function (quantitative: Simpson, Teicholz) Diastolic function (quantitative) Contraction pattern (quantitative) Valvular disease (quantitative)	Size (quantitative: diameter and wall thickness) Systolic function (quantitative: MAPSE, aortic VTI)
Right ventricle	Size (qualitative) Systolic function (quantitative: TAPSE, RV/LV ratio) Valvular disease (qualitative: colour doppler)	Size (quantitative) Valvular disease (quantitative)	
Inferior vena cava	Size (quantitative) Respiratory variation (quantitative)		
Procedures			Pericardiocentesis
**Thoracic ultrasound**			
Syndromes	Consolidation** Pleural effusion Interstitial syndrome*** Pneumothorax		
Procedures	Pleural effusion drainage (thoracentesis and/or intercostal drain insertion)		Tracheostomy
**Diaphragm ultrasound**			
		Thickness Thickening fraction	Excursion
**Abdominal ultrasound**			
	Free fluid Bladder volume (qualitative) Hydronephrosis (qualitative)****	Liver and biliary tree (cholecystitis) Renal resistive index Hydronephrosis (quantitative)	Aorta
Procedures	Ascites drainage		
**Vascular ultrasound**			
Syndromes	DVT (proximal 3-point compression)*****	DVT (Doppler)	
Vascular access	Femoral vein Jugular vein Radial artery Femoral artery		Subclavian vein
**Other modalities**			
		Nerve block Muscle Skin and soft tissue Optic nerve sheath diameter Airway management	Transcranial Doppler

*LV* left ventricle, *RV* right ventricle, *MAPSE* mitral annulus plane systolic excursion, *VTI* velocity time integral, *TAPSE* tricuspid annulus plane systolic excursion, *DVT* deep vein thrombosis

*Post-cardiac arrest care was perceived to have no specificities; most of the features are covered by assessment of hypovolemia/right ventricle/left ventricle/tamponade/severe valvular dysfunction as reported in the left column

**Consolidation refers to different pulmonary conditions characterized by different degrees of loss of aeration and increase in density, such as infection, contusion, infarction or atelectasis [8]

***Interstitial syndrome refers to a collection of conditions affecting the lung interstitium characterized by increased B-lines generated by juxtaposition of alveolar air and septal thickening (from fluid or fibrosis) [8]

****Qualitative measurement refers to yes/no answer

*****Three-point compression method involves compression at (1) common femoral vein and saphenofemoral junction, (2) popliteal vein and (3) mid-thigh level

## Discussion

This manuscript offers the most up-to-date set of core CCUS competencies for an intensive care clinician. It must be emphasized that the competencies which were not included or where no consensus could be reached do have clinical merit and use. The agreed core competencies should form the foundation for further learning and focus primarily on qualitative analysis and assessment. The group recommends that intensivists should be encouraged to develop their CCUS skillsets beyond the core competencies in a structured fashion.

Another important consideration for competency inclusion is that the skill needs to be deliverable and useful to the general intensivist. More specialized units require more specific (advanced) skill sets, such as transcranial Doppler in neurocritical care units.

For purposes of this statement, the core competencies have been listed by organ system. While the listed competencies describe identification of the relevant abnormality using CCUS, we emphasize that competency also requires that the intensivist has mastery of the cognitive base required to integrate the CCUS findings into the clinical management plan, i.e. it does not suffice for the intensivist to simply acquire an adequate image. The intensivist must also be able to integrate the results into a holistic, whole-body approach at the bedside. As an example, lung, cardiac and vascular ultrasound scans may all be indicated and performed in a patient with suspected pulmonary embolism.

The most common reason for not including a CCUS competency into the list was that some competencies were considered to be too specialized in nature or impractical as part of core training (Supplementary material 2). For example, although the use of Doppler-based measurements is acknowledged as an important technique, the consensus of the core group was that such quantitative methods should be considered to be part of advanced CCUS. The distinction between basic and advanced components of CCUS is analogous to that presented in previous ESICM statements on CCUS by the expert panel [1, 2] that established a clear distinction between basic and advanced critical care echocardiography.

This document does not address the challenging issue of how to deliver effective training programmes both for critical care trainees and attending level intensivists who need to develop competency in CCUS. This will be key to fostering full integration of CCUS into frontline critical care practice as a pan-European and international standard.

Some limitations of the modified Delphi method should be considered [9]. The most important one is the loss of subject anonymity during the face-to-face meeting, but on the other hand, absence of this meeting may deny the experts the necessary clarification of reasons for disagreement. It is also important to note that this method contains some methodological problems such as the bias in the selection of participants; in our case, the greater proportion of participants was from Europe. In our study, 12 of the 15 experts attended this last meeting, so results could have been biased in favor of the experts in attendance. This bias was minimized however, by distribution of the results to the whole group for final remarks.

This document was approved by all panel members and endorsed by the ESICM. While the document has been developed for standard setting within Europe, the representation by international societies from North America, ANZAC and Asian countries in our core group suggests broader, worldwide utility is plausible.

## Conclusion

In conclusion, these recommendations offer the most contemporary and evolved set of core CCUS competencies for an intensive care clinician yet described. Given the continual evolution of understanding and broadening use of ultrasound in intensive care medicine, these recommendations are but an instantiation of a dynamic, iterative process. With an ever increasing number of ultrasound practitioners, such guidance will ensure a high standard of training and hence patient care.

## Supplementary information

**Additional file 1.** List of contributors

**Additional file 2.** Competencies which achieved agreement for exclusion from core CCUS list

## Data Availability

Not applicable
