# Supplementary material for: Recommendations for core critical care ultrasound competencies as a part of specialist training in multidisciplinary intensive care: a framework proposed by the European Society of Intensive Care Medicine (ESICM)
Source: Crit Care. 2020 Jul 3;24:393. doi: 10.1186/s13054-020-03099-8 (PMC7333303; doi:10.1186/s13054-020-03099-8)
Supplement: Supplementary file 1 — Additional file 1. List of contributors [file 13054_2020_3099_MOESM1_ESM.docx]

**Supplementary material 1**

**List of contributors**

- Hans Ulrich Rothen, Dept of Intensive Care Medicine, Bern University Hospital—Inselspital, Bern, Switzerland
- Fabio Silvio Taconne, Dept of Intensive Care, Erasme Hospital, Université Libre de Bruxelles (ULB), Brussels, Belgium
- Armand Mekontso Dessap, Service de Réanimation Médicale, CHU Henri Mondor, Université Paris Est Créteil, Créteil, France
- Ib Jammer, Dept of Anesthesia and Intensive Care, Haukeland University Hospital, Bergen, Norway
- Daniele G. Biasucci, Dept of Intensive Care Medicine and Anesthesiology, “A. Gemelli” University Hospital Foundation, Catholic University of the Sacred Heart, Rome, Italy
- Filipe Gonzalez, Dept ofIntensive Care, Hospital Garcia de Orta, Almada, Portugal
- Balan Cosmin, Dept of Anaesthesiology and Intensive Care, Carol Davila University of Medicine and Pharmacy, Bucharest, Romania
- Hynek Riha, Dept of Anaesthesia and Intensive Care, Institute for Clinical and Experimental Medicine, Prague, Czech Republic
- Christoph Ganter, Institute of Intensive Care Medicine, University Hospital Zürich, Zurich, Switzerland
- João Santos Silva, Dept of Intensive Care, Hospital Santa MariaCentro Hospitalar Universitario Lisboa NorteLisbonPortugal
- Lucia Lopez-Rodriguez, Dept of Intensive Care, Hospital Universitario de Getafe, Getafe, Spain
- Miguel Tavares, Dept of Intensive Care, Santo António General Hospital, Porto, Portugal
- Jan Benes, Department of Anesthesiology and Intensive Care Medicine, Faculty of Medicine in Plzeň, Charles University, Plzeň, Czech Republic
- Gernot Gorsewski, Dept of Anaesthesia and Intensive Care, State Hospital Feldkirch, Feldkirch, Austria
- Jesús-Andrés Álvarez-Fernández, Dept. Intensive Care, Hospital Universitario de Getafe, Getafe, Spain
- Sara Sher, Dept of Intensive Care, Ospedale Niguarda Ca’Granda, Milan, Italy
- Reto Etter, Dept of Intensive Care, Spitalzentrum Biel, Biel, Switzerland
- Peter Faybik, Dept of Anesthesia, Intensive Care, and Pain Therapy, Medical University of Vienna, Austria
- Michelle Chew, Dept of Anesthesiology and Intensive Care, Medical and Health Sciences, Linköping University, Linköping, Sweden
- Thomas Hamp, Dept of Anesthesia, Intensive Care, and Pain Therapy, Medical University of Vienna, Austria
