# Supplementary material for: Recommendations for core critical care ultrasound competencies as a part of specialist training in multidisciplinary intensive care: a framework proposed by the European Society of Intensive Care Medicine (ESICM)
Source: Crit Care. 2020 Jul 3;24:393. doi: 10.1186/s13054-020-03099-8 (PMC7333303; doi:10.1186/s13054-020-03099-8)
Supplement: Supplementary file 2 — Additional file 2. Competencies which achieved agreement for exclusion from core CCUS list [file 13054_2020_3099_MOESM2_ESM.docx]

**Supplementary material 2**

**Competencies which achieved agreement for exclusion from core CCUS list**

Echocardiography

The potential role and echocardiographical measurements that can be performed are continuing to expand. The initial consensus statements were clear that core echocardiography assessment should be qualitative in nature.

Quantitative assessment along with Doppler techniques were mostly excluded as the added complexity requires extensive knowledge beyond the remit of core CCUS - the old adage of ‘a little knowledge is a dangerous thing’ rings true in this situation.

The group discussed the use of colour Doppler in the assessment of valvular pathologies such as mitral regurgitation, however, it only constitutes part of the assessment. International guidelines from cardiological societies (ref) place heavy emphasis on several quantitative measures for each valve being assessed.

The exception to quantitative assessment is the use of M-mode to measure the Tricuspid Annular Plane Systolic Excursion (TAPSE) as a measure of right ventricular systolic function. It is well validated and with good intra- and inter-observer reproducibility.

Thoracic

There was consensus that ultrasound assessment of the diaphragm should not be included as a core competency due to the significant intra- and inter-observer variability when measuring diaphragm thickness and thickening.

Abdomen

Ultrasound assessment of the liver, biliary tree and spleen was not included. These skills were felt to be beyond the remit of a general intensivist in the acute setting.

Other modalities

Other modalities, including ultrasound assessment of the nervous system, musculoskeletal system and airway were also included in the discussion to broaden the spectrum of clinical utilities being considered. These were considered too specialised to be included as core competencies. Ultrasound for the airway management is increasing its applications but it requires more advanced knowledge, however lung ultrasound could be used for assessing endotracheal tube position.

**Competencies where consensus could not be reached**

These competencies required the most deliberation as there is no denying their clinical applicability. The individual reasons for their eventual exclusion are discussed below. The group sought to provide a pragmatic balance of clinical utility with training requirements and delivery. With this in mind, the group felt that after the core competencies, it is this group of skills that colleagues should focus on.

Echocardiography

Quantification of right and left ventricular size was extensively discussed. Accuracy of these measurements require that the images obtained are not foreshortened and correct use of the cursor to outline the ventricular cavity, both of which require practice and experience.

The ability to measure aortic velocity time integral (VTi) was also enthusiastically debated in view of its clinical applicability/usefulness as a marker of fluid responsiveness and as a haemodynamic parameter. However, this was excluded as it relied on Doppler techniques which are not part of core CCUS.

MAPSE was also discussed, whilst easy to acquire, it is still not properly validated; the cut-off for normal values is not well established and it is not included in international guidelines.

Ultrasound guided pericardiocentesis is without doubt, invaluable to increasing the success and safety profile of the procedure. However, the group could not reach a consensus because pericardiocentesis is an uncommon procedure and there was concern about the availability of training opportunities to achieve competency.

Thoracic

There was no consensus reached with regards to diaphragmatic excursion. Whilst the measurement of diaphragmatic excursion is more straightforward and reproducible compared to diaphragmatic thickening, it is considered less clinically reliable (especially in context of predicting weaning from mechanical ventilation) compared to thickening and thickening fraction.

Due to issues similar to those regarding pericardiocentesis, the use of ultrasound to guide percutaneous tracheostomy could not reach consensus.

Abdomen

No consensus could be reached for assessment of the aorta. Whilst a rather straightforward assessment, it is less commonly encountered in the critical care setting (as opposed to the emergency department).

Other modalities

Of all the ultrasound techniques used for the nervous system, transcranial doppler is perhaps the most established and frequently studied. Whilst there is little doubt regarding its usefulness, the group felt that this is an advanced technique, with clinical utility limited to specialist units rather than general ICUs.

## Supplementary References

1. American College of Chest Physicians Critical Care Ultrasonography Programme.<http://www.chestnet.org/Education/Advanced-Clinical-Training/Certificate-of-Completion-Program/Critical-Care-Ultrasonography>.
2. Frankel HL, Kirkpatrick AW, Elbarbary M. Guidelines for the Appropriate Use of Bedside General and Cardiac Ultrasonography in the Evaluation of Critically Ill Patients-Part I: General Ultrasonography. Crit Care Med. 2015; 43(11): 2479–2502.
3. Pustavoitau A, Blaivas M, Brown SM, et al. From the Ultrasound Certification Task Force on behalf of the Society of Critical Care Medicine: Recommendations for achieving and maintaining competency and credentialing in critical care ultrasound with focused cardiac ultrasound and advanced critical care echocardiography. Official Statement from Society of Critical Care Medicine. <https://journals.lww.com/ccmjournal/Documents/Critical%20Care%20Ultrasound.pdf>
4. UK Core Ultrasound Skills in Intensive Care CUSIC programme.<http://www.ics.ac.uk/ics-homepage/accreditation-modules/cusic-accreditation/>
5. UK Focussed Intensive Care Echocardiography FICE programme.<http://www.ics.ac.uk/ics-homepage/accreditation-modules/focused-intensive-care-echo-fice/>
6. Arntfield R, Millington S, Ainsworth C, et al. Canadian recommendations for critical care ultrasound training and competency. Can Respir J. 2014; 21(6): 341–345.
7. Michels G, Zinke H, Möckel M, et al. Empfehlungen zur Ultraschallausbildung in der internistischen Intensiv- und Notfallmedizin: Positionspapier der DGIIN, DEGUM und DGK. Medizinische Klinik - Intensivmedizin und Notfallmedizin 2017; 112 (4): 314-319.
8. Slegers CAD, Blans MJ, Bosch FH. Instructions for the use of critical care ultrasound in Dutch daily practice: the Rijnstate ICU manual, ready for broad acceptance? Neth J Crit Care June 2014; 18(3): 4-18.
9. Wytyczne Polskiego Towarzystwa Anestezjologii I Intensywnej Terapii W Zakresie Uzyskiwania Akredytacji W Zakresie Echokardiografii I Ultrasonografii. http://www.anestezjologia.org.pl/
10. POCUS program Schweizerischen Gesellschaft für Ultraschall in der Medizin <http://www.sgum.ch/weiterbildung_fa/dokumente/fa_pocus_f.pdf/>
11. Lichtenstein D, Goldstein I, Mourgeon E, et al. Comparative diagnostic performance of auscultation, chest radiography, and lung ultrasonography in acute respiratory distress syndrome. Anesthesiology 2004; 100: 9-15.
12. Lichtenstein D and Meziere G. Relevance of lung ultrasound in the diagnosis of acute respiratory failure – the BLUE protocol. Chest 2008; 134: 117-125.

World Interactive Network Focused on Critical Care Ultrasound (WINFOCUS). http://winfocus.com
